# Analysis of Individual Protein Regions Provides Novel Insights on Cancer Pharmacogenomics

**DOI:** 10.1371/journal.pcbi.1004024

**Published:** 2015-01-08

**Authors:** Eduard Porta Pardo, Adam Godzik

**Affiliations:** Program on Bioinformatics and Systems Biology, Sanford-Burnham Medical Research Institute, La Jolla, California, United States of America; University College London, United Kingdom

## Abstract

The promise of personalized cancer medicine cannot be fulfilled until we gain better understanding of the connections between the genomic makeup of a patient's tumor and its response to anticancer drugs. Several datasets that include both pharmacologic profiles of cancer cell lines as well as their genomic alterations have been recently developed and extensively analyzed. However, most analyses of these datasets assume that mutations in a gene will have the same consequences regardless of their location. While this assumption might be correct in some cases, such analyses may miss subtler, yet still relevant, effects mediated by mutations in specific protein regions. Here we study such perturbations by separating effects of mutations in different protein functional regions (PFRs), including protein domains and intrinsically disordered regions. Using this approach, we have been able to identify 171 novel associations between mutations in specific PFRs and changes in the activity of 24 drugs that couldn't be recovered by traditional gene-centric analyses. Our results demonstrate how focusing on individual protein regions can provide novel insights into the mechanisms underlying the drug sensitivity of cancer cell lines. Moreover, while these new correlations are identified using only data from cancer cell lines, we have been able to validate some of our predictions using data from actual cancer patients. Our findings highlight how gene-centric experiments (such as systematic knock-out or silencing of individual genes) are missing relevant effects mediated by perturbations of specific protein regions. All the associations described here are available from http://www.cancer3d.org.

## Author Summary

There is increasing evidence that altering different functional regions within the same protein can lead to dramatically distinct phenotypes. Here we show how, by focusing on individual regions instead of whole proteins, we are able to identify novel correlations that predict the activity of anticancer drugs. We have also used proteomic data from both cancer cell lines and actual cancer patients to explore the molecular mechanisms underlying some of these region-drug associations. We finally show how associations found between protein regions and drugs using only data from cancer cell lines can predict the survival of cancer patients.

## Introduction

With the body of genomic and pharmacologic data on cancer growing exponentially, the main bottleneck to translate such information into meaningful and clinically relevant hypothesis is data analysis [1]–[3]. While numerous methods have been recently applied to the analysis of such datasets [4] most of them, particularly those dealing with mutation data [5], use a protein-centric perspective, as they do not take into account the specific position of the different mutations within a protein [6], [7]. Such approaches have been proven useful in many applications; however, they cannot fully deal with situations in which different mutations in the same protein have different effects depending on which region of the protein is being altered [8]. This idea can be easily explained by the fact that most proteins are modular, consisting of several distinct domains and/or functional regions, which we collectively call PFRs (protein functional regions) here. For instance, a receptor tyrosine kinase, such as EGFR, has two PFRs - an extracellular region, which is responsible for the interaction with the ligand or with other receptors, and an intracellular kinase domain, which in turn is responsible for the phosphorylation of its substrates. A phenotype, such as the response towards a drug, can be influenced by alterations of proteins at the whole-protein level (changes in expression, deletion or epigenetic silencing of a gene), but also changes, such as mutations, modifying only the extracellular or the kinase domains. More importantly, even though it is likely that each of the three types of alterations (whole-protein, only in the extracellular region or only in the kinase domain) will have different consequences [9], only those involving the whole protein have been studied.

To explore how perturbations of specific PFRs in different proteins might influence the sensitivity of cancer cell lines towards specific drugs we developed a novel algorithm called e-Drug. This algorithm analyses patterns of mutations in functional regions within each protein in the human proteome and identifies those associated with changes in the activity of anticancer drugs. Our definition of PFRs includes protein domains, both those present in Pfam database and those predicted to exist using our in-house tools, and intrinsically disordered regions. Similar approaches focusing on Pfam protein domains have been used previously to study the molecular mechanisms underlying the pleiotropy of certain genes, especially those related to Mendelian disorders [10], [11], and cancer [12]–[14]. In the context of the analysis of drug-related data, PFRs have been mainly used to study phenomena such as polypharmacology or the structural details underlying interactions between drugs and domains [15], [16]. However, to the best of our knowledge, such PFR-centric analyses have ever been used to study cancer pharmacogenomic datasets.

## Results

### Analysis schema and overall results

**Figure 1:**
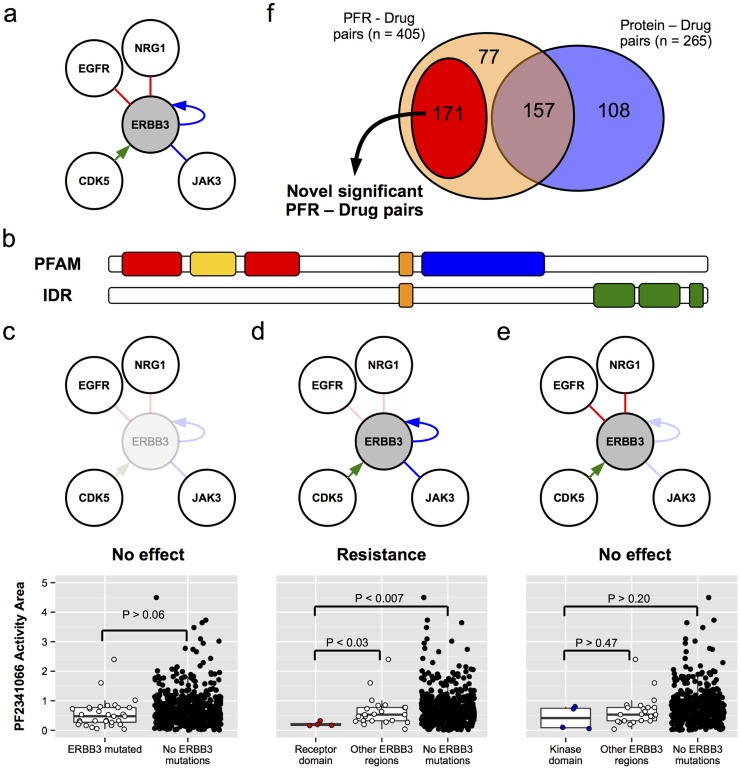
Analysis at the functional region level allows us to gain novel insights from pharmacogenomics data. (a, b) Mapping of the different ERBB3 functions to specific regions of the protein. Each functional relationship can be associated to a specific domain or intrinsically disordered region in ERBB3. For example, EGF receptor domains (red boxes in (b)) mediate physical interactions between ERBB3 and EGFR and NRG1 (red edges in (a)). (c) Methods focusing at the whole-protein level can not find any association between ERBB3 mutations and the activity of PF2341066. (d) Mutations altering specifically the N-terminal EGF receptor are associated to lower drug activity. (e) Mutations affecting another PFR in ERBB3, its kinase domain, and that, thus, are mainly affecting other functional regions, are not associated to any changes in drug activity. (f), Venn diagram showing the different thresholds that we have established in order to minimize false positives. We only kept PFRs with (I) p<0.001 when compared to cell lines with no mutation in the protein, (II) p<0.05 when compared to cell lines with mutations in other regions of the same protein and (III) with p>0.01 at the protein level.

The e-Drug analysis protocol introduced here is illustrated in Fig. 1 on the example of the ERBB3 protein and the c-Met inhibitor PF2341066. Some of the many functional relationships of this protein include physical interactions (with EGFR, NRG1 and JAK3) or phosphorylations (by CDK5 or ERBB3 itself). Each of these relationships can be mapped to a specific PFR within ERBB3. For example, the N-terminal EGF receptor domains (shown in red in Fig. 1) mediate the interactions with EGFR and NRG1, whereas ERBB3's kinase domain (shown in blue in Fig. 1) interacts with JAK3 and phosphorylates other ERBB3 molecules.

When using the protein level analysis, cell lines with mutations in ERBB3 do not show any bias in the activity of PF2341066, leading to a wrong conclusion that mutations in this protein do not influence the sensitivity towards this drug. However, the PFR level analysis shows that cell lines with mutations in the first receptor domain are resistant to treatment with the PF2341066 inhibitor, while those with mutations in any other PFRs of this protein, such as the kinase domain of the second receptor domain, do not show any specific behavior.

Following this protocol, we have identified 171 statistically significant PFR-drug associations (p<0.05 in the comprehensive, multistage significance analysis as described in the Methods Section and S1 Supporting Material). The full list is provided in the S1 Table and is available on-line from a newly developed resource at http://cancer3d.org [17].

**Figure 2:**
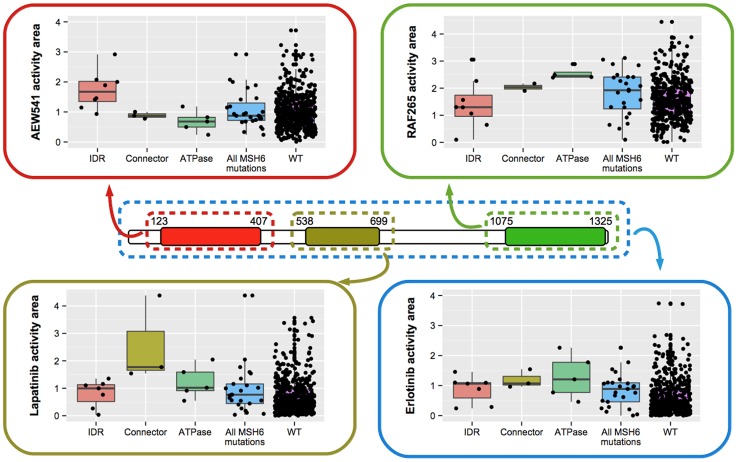
Perturbations of different regions in the same protein can have different drug effects. (a) Missense mutations in different PFRs of MSH6 lead to increased sensitivity towards three different drugs: AEW541, RAF5 and Lapatinib. The protein level analysis on the other hand reveals a potential association with Erlotinib (shown in blue). This highlights the complementarity between protein and PFR-centric approaches.

We have found some cases where two PFRs from the same protein are associated with different drugs. For example, the MSH6 protein contains 3 different PFRs associated with 3 different drugs (Fig. 2). Mutations in the N-terminal IDR are associated with increased AEW541 activity, while mutations in the connector (PF05188) and ATPase (PF00488) domains are associated with higher Lapatinib and RAF265 activities, respectively. Given that MSH6 has been recently shown to be involved in pathways related to the repair of DNA-double-strand breaks [18], the association identified here between mutations in MSH6's ATPase domain, as well as other PFRs in PAXIP1 or TP53, and the activity of RAF265 suggest that the DNA-damage response pathway might have a role in modulating the activity of this drug.

### Integration of CCLE with other molecular datasets provides further insights into the role of individual PFRs

**Figure 3:**
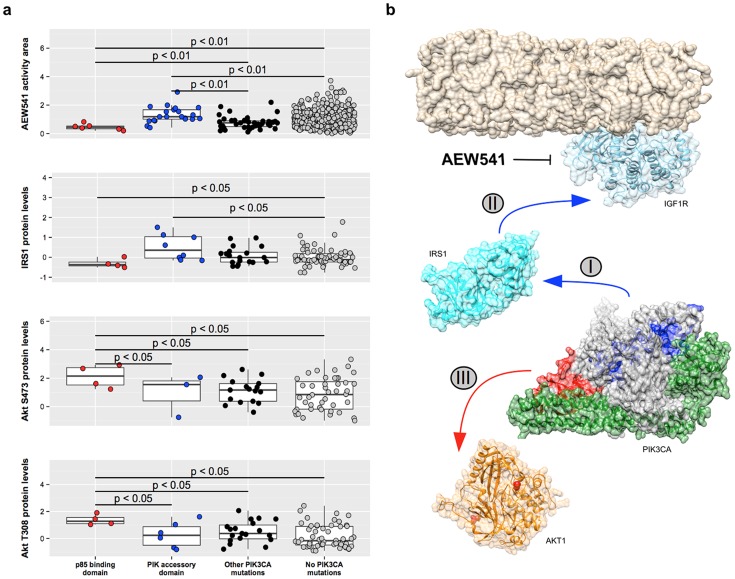
Using complimentary datasets to validate some of the predictions by e-Drug. (a) Missense mutations in PIK3CA can have opposite effects in terms of AEW541 activity depending on the PFR affected. Mutations in the p85-binding and PIK accessory domains are associated with lower and higher drug activities respectively (upper panel). By integrating our analysis with proteomics data from TCPA we have been able to propose a mechanism for that. It appears that IRS1 protein expression is lower in cells with p85-binding mutations, but higher in those with PIK mutations (second panel). Moreover, Akt1 phosphorylation levels are higher in cell lines with p85-binding domain mutations (two lower panels). (b) Proposed mechanisms for the two PFR-AEW541 associations. AEW541 inhibits the kinase domain of IGF1R (upper blue protein). In those cell lines with mutations in the PIK domain of PIK3CA (shown in blue PIK3CA's structure), there is a gain of interaction between this protein and IRS1 (I). This will likely increase the signaling through IGF1R (II), explaining why cell lines with mutations in this domain are more sensitive to the inhibition of this receptor. On the other hand, cell lines with mutations in the p85-binding domain (shown in red in PIK3CA's structure) have lower IRS1 expression and higher AKT1 phosphorylation levels. Together, this suggests that PIK3CA is active in this cell lines independently of its interaction with extracellular receptors, signaling directly downstream towards AKT1 (III). This would explain why these cells are resistant to AEW541.

The best examples of the advantages of studying mutation effects on individual PFRs are those where mutations in different regions of the same protein are associated with the same drug but in opposite directions. This is the case of PIK3CA and the IGF1R inhibitor AEW541. Using e-Drug we found that mutations in the p85 binding domain (PF02192) decrease the activity of the AEW541 while mutations in the PIK accessory domain (PF00613) are associated with increased activity of the same drug (Fig. 3). Mutations in different regions of PIK3CA are known to be oncogenic through different molecular mechanisms [19], which could also explain the opposite effects in AEW541 sensitivity observed for these two domains.

To find features that could explain the different responses to AEW541 depending on the PIK3CA domain mutated, we used proteomics data from The Cancer Proteome Atlas [20]. We focused our analysis on IRS1 expression levels as well as Akt phosphorylation status in the cell lines with mutations in the two PIK3CA domains, because these proteins are immediately up and downstream from PIK3CA, respectively (Fig. 3).

Cell lines with mutations in the PIK accessory domain did not have changes in the phosphorylation levels of Akt at neither T308 (p>0.34) nor S473 (p>0.07), but did have higher IRS1 expression (p<0.05). These results agree with recent data showing that the E545K mutation in PIK3CA enhances its interaction with IRS1 [21]. Since IRS1 mediates the interaction between IGF1R and PIK3CA, this increased interaction with IRS1 (and therefore dependence on interaction with receptor tyrosine kinases such as IGF1R) could explain why cell lines with mutations in PF00613 are more sensitive to IGF1R inhibition (Fig. 3).

On the other hand, cell lines with mutations in the p85 binding domain showed higher Akt phosphorylation levels at both T308 (p<0.01) and S473 (p<0.02), and also had lower IRS1 protein levels (p<0.01). Since Akt is one of the main PIK3CA effectors, a possible interpretation of these results is that cell lines with mutations in the p85-binding domain have intrinsically active PIK3CA phosphorylation activity, regardless of its interaction with receptor tyrosine kinases such as IGF1R. In this scenario, inhibiting IGF1R with AEW541 would have little effects, as these cells are already signaling downstream towards Akt (Fig. 3).

Finally, given recent concerns about pharmacogenomic data using cell lines [22] we compared these results to those obtained from data on human tumors analyzed by TCPA (n = 2229). We confirmed all the tumors with mutations in PF02192 have higher levels of Akt phosphorylation at both T308 and S473. The same samples also have lower IRS1 levels than those with no mutations in PF00613 or no mutations at all. Tumor samples with mutations in PF00613, on the other hand, have higher IRS1 levels and no changes in Akt phosphorylation status.

### Drug-PFR correlations predict success of cancer treatment

**Figure 4:**
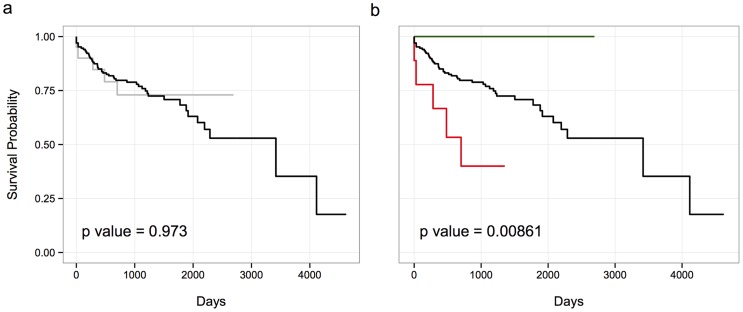
PFR perturbations identified using data from cell lines predict the survival of patients treated with Irinotecan. (a) Proteins with PFR associated to Irinotecan resistance can not be used to successfully stratify cancer patients treated with this drug, as there are no differences between patients with mutations in such proteins (gray) and those without them (black) (b) Specific PFR in these proteins do predict the outcome of cancer patients. Patients with mutations altering the PFRs found using CCLE (red) have worse outcomes that those with mutations in other regions of the same protein (green) or no mutations (black).

Since we had been able to confirm the hypothetical molecular mechanisms underlying the PFR-drug associations between AEW541 and PIK3CA in tumor samples, we wondered whether we could also predict survival of actual cancer patients using the PFRs identified in the CCLE data. To that end, we used clinical data from patients whose tumors have been analyzed by The Cancer Genome Atlas (TCGA) groups [23] to find patients that had been treated with drugs included in the CCLE. Since most of these drugs are still under clinical research, there were sufficient data only to analyze two types of drugs: Paclitaxel (n = 778) and the topoisomerase inhibitors Irinotecan and Topotecan (n = 188). We used genomic data of the patients to find those who had mutations in PFRs that are associated to increased resistance towards these drugs in our analysis (Fig. 4). While we found no differences in patients treated with paclitaxel (p>0.9), patients that had mutations in PFRs associated with resistance to Topoisomerase inhibitors had worse outcomes (p<0.01) than those with mutations in other regions of the same proteins or no mutations in these proteins at all. Interestingly, the mutation status of the whole proteins that contain the associated PFRs cannot predict the outcome of the patients (p>0.9), suggesting that only mutations in the specific PFRs, but not in other regions of the same proteins, confer resistance to topoisomerase inhibitors.

### Proteins and PFRs associated with drugs do not usually overlap with drug targets

One of the possible mechanisms for a PFR to be associated with differential drug activity is that the protein itself directly interacts with the drug of interest. To explore this hypothesis, we compared the set of proteins associated with each drug at both whole-protein and individual PFR levels, to the set of drug targets as identified by the STITCH database [24]. Of the 19 drugs that had at least one known target, only AZD6244 had its associated proteins and PFRs enriched with its targets, as mutations in two of the five genes known to code for proteins interacting directly with the drug, BRAF and KRAS, are also associated with differential activity for this drug (p<0.005). Expanding our search by varying the STITCH interaction score, including proteins that interact with compounds that have similar structures to the drugs included in the analysis (Tanimoto score >0.70) or to proteins interacting with the drug targets also did not show any statistically significant associations (S4 Fig.).

### Gene set enrichment analysis of PFRs and proteins that correlate with drug activity reveals common functions

**Figure 5:**
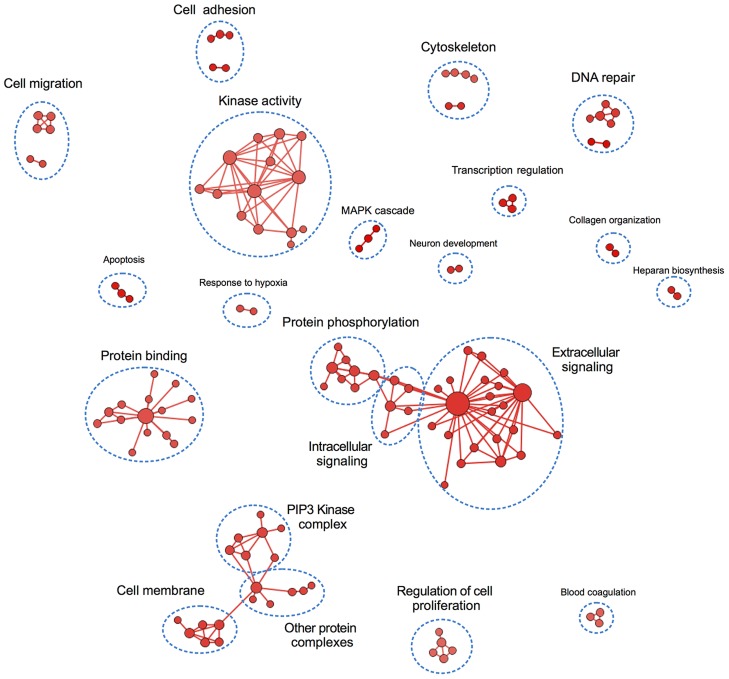
Enrichment map of the proteins associated with differential drug activity at both, whole-protein and individual region levels. We performed a gene-set enrichment analysis by comparing Gene Ontology (GO) annotations of the 316 proteins associated with different drugs at both levels of resolution (whole-protein and individual PFRs) against the whole human genome. All the GO terms identified here showed an enrichment in the biomarker group, and most of them relate to pathways and functions associated with carcinogenesis, metastasis, and drug resistance, such as regulation of cell proliferation, kinase activity, cell migration, cell adhesion, MAPK cascade, or response to hypoxia. In the figure, GO terms are connected when they are related according to the gene ontology.

We did a gene set enrichment analysis using GO annotations downloaded from Uniprot to understand the shared functions and relationships of the proteins and regions associated with changes in drug activity, (Fig. 5). Several groups of GO terms identified in this analysis, such as those related to signaling cascades (extracellular and intracellular signaling), signal transduction (kinase activity or protein phosphorylation), or protein binding, suggest that these genes might be involved in either the same pathways as the actual drug targets or similar pathways that might have some level of redundancy. Other GO terms, such as apoptosis, regulation of cell proliferation, or response to hypoxia, are functions known to play a role in drug resistance and carcinogenic potential of cells.

Another group of GO terms identified in our analysis are those associated with the cytoskeleton. Given that most of the drugs analyzed in this study (17 out of 24) are kinase inhibitors, this was an unexpected observation. However, there is some evidence of the relationship between cytoskeleton proteins and the activity of kinase inhibitors in the literature. For example, many receptor tyrosine kinases, such as EGFR, HER2, IGF1R, or FGFR, undergo internalization upon ligand binding. Moreover, interactions between Erlotinib and MYO2 or MYH9 have been described, and a MYH9 inhibitor synergizes with EGFR inhibitors to induce apoptosis in cells carrying the drug-resistant mutation T790M [25].

## Discussion

Identifying biological features that correlate with the activity of anticancer drugs has been the subject of a significant and growing research focus in recent years. However, most of these efforts do not take into account the modular nature of proteins and focus on perturbations at the whole-protein level. Such analyses are likely to miss cases in which the location of the mutation within the protein influences its effects. Here we have described what is, to the best of our knowledge, the first systematic analysis of drug activity associations that distinguishes between different functional regions within proteins. We have shown that by focusing on specific PFRs we can find 171 associations between mutations in specific protein regions and changes in the activity of anticancer drugs. These associations could not have been identified by protein-centric approaches, as cell lines carrying mutations in other PFRs of the same protein (i.e. perturbing regions that mediate other functions) are not associated with any, with different or sometimes the opposite drug phenotypes.

Cases in which the same gene is associated with different drugs through different PFRs, as in the case of MSH6 and the kinase inhibitors Erlotinib, AEW541, Lapatinib, and RAF265 can provide insights into the mechanisms of the drug pleiotropy of a given gene, aiding in further experiments. A variation of this category is the association between PIK3CA and the AEW541, where mutations in different PFRs can have opposing effects in terms of the activity of the drug.

We have also shown the practical value of the PFR-drug associations discovered here on the independent data from the TCGA consortium. Specifically, we have shown that patients from the TCGA harboring mutations in regions associated with resistance to the drugs used to treat them have lower survival rates than patients with mutations in the very same genes but in regions not showing any association to the activity of such drugs. This result not only provides evidence to the significance of our approach, but it also argues in favor of the value of drug activity data collected using cell lines (at least in the case of the CCLE), an issue that has recently drawn significant attention [22] and that will probably require substantial work in the near future in order to be solved.

Another interesting result of the analysis presented here is that the proteins identified in our analysis as modulating sensitivity of cancer cells to drugs, are distinct from the actual targets of these drugs nor interact directly with them. This observation suggests that these genes modify drug activity through indirect interactions. For example, mutations in genes related to the cytoskeleton (a subset enriched in the genes identified in our analysis) might alter the potency of kinase inhibitors by changing the trafficking pattern of receptor tyrosine kinases. This is one of the most unexpected findings in our analysis. While more analyses and direct experimental verifications of these correlations are needed, such relations may suggest targets for therapies sensitizing cancer cells to chemotherapy with specific drugs.

Overall, this work expands the number of correlations between cancer somatic mutations and drug activity, thus increasing the information we can extract from every dataset. Focusing on PFRs, corresponding to protein domains or IDRs, provides better statistical results than analysis of individual mutations and allows to identify correlations in cases where different effects cancel out and thus are missed on the whole gene analysis level. At the same time, it provides more details about the mechanism of the drug resistance than the analysis on the gene level. Increasing the number and details of features that predict the activity of anticancer drugs has important consequences in the field of personalized medicine, as it increases accuracy in stratifying patients into groups that require different treatment regiments and can suggest drug combinations as exemplified for EGFR and MYH9.

One interesting direction of work that we have not been able to address refers to the interaction between multiple drug activity modifiers. Now that we have been able to extend the catalog of alterations that alter a cell's sensitivity towards a drug using our PFR-centric approach, what happens when there are multiple such alterations in the same cell line or patient? Do they cancel each other if they have opposite effects? Do they synergize if they point towards the same direction? Most attempts to answer these challenging questions are based on machine learning approaches [5] which, given the multidimensional nature of the problem, seems to be the most natural approach. However, simple methods based on naively counting the presence or absence of specific alterations, such as our own analysis of TCGA clinical data for Irinotecan and Topotecan presented here or analyses based on synthetic lethal interaction networks [4], seem to also have some predicting power. Regardless of the specific approach, these are questions that will need to be answered in order to achieve the promise of personalized medicine.

Another generalization of our results is that data obtained using gene knockouts, silencing RNAs, or other technologies that completely abolish the activity of individual proteins might miss more subtle effects caused by modifications of specific PFRs. Finally, we would like to emphasize that, just like the analyses at the protein level is not limited to the identification of features that correlate with drug activity, the analysis of PFR perturbations can be useful when looking for features associated with any phenotype.

## Materials and Methods

### Cell line mutations

We have used the CCLE dataset, which includes the mutation profiles of 1,668 genes in 906 human cancer cell lines and drug activity data for 24 different anticancer compounds. We focused our analysis on missense mutations, as truncating mutations can sometimes be misleading when performing the analysis in terms of functional regions. For example, when analyzing a protein that contains two different domains, if a truncating mutation happens in the first domain, it is not obvious whether the functional consequences of the mutation are caused by the fact that the first domain is altered or that the second domain is missing. We mapped the missense mutations reported by CCLE from their genomic coordinates to every protein coding isoform from ENSEMBL using the Variant Effect Predictor tool [26]. From the original 42,603 genomic-point mutations in 1,668 genes, we obtained 156,817 protein missense mutations in 9,311 proteins.

### Drug activity data

The CCLE contains data on the drug activity of 24 different compounds in 479 cell lines from 8-point dose-response curves. These curves are adjusted to a logistical-sigmoidal function and described by 4 different variables: the maximal effect level (Amax), the drug concentration at half-maximal activity of the compound (EC50), the concentration at which the drug response reached an absolute inhibition of 50% (IC50), and the activity area, which is the area above the dose-response curve. In our analysis we have used only the activity area because, according to the CCLE, it captures simultaneously both variables of drug activity: its efficacy and its potency.

### Protein functional regions

We defined protein functional regions as domains or intrinsically disordered regions. We decided to include intrinsically disordered regions because these can also contain important functional regions such as phosphorylation sites or regions that regulate or mediate protein interactions [27]. To identify protein domains, we retrieved, for each protein isoform, annotated Pfam domains from ENSEMBL. We have also included a set of 1,300 novel potential domains identified by AIDA, an algorithm based on iterative recognition of domains by homology recognition algorithms with various sensitivities [28]. We used Foldindex [29] to predict intrinsically disordered regions for each protein, including in our analysis those regions with a predicted unfolded score below –0.1.

Finally, we mapped the different mutations of each cell line to these protein features, giving us a total of 30,798 altered regions in 906 cell lines. These regions are divided into 19,918 Pfam domains and 10,880 intrinsically disordered regions. Note that the features can overlap, as the predictions were performed independently and there is no reason why, for example, an intrinsically unfolded region cannot overlap with (or even be located within) a Pfam domain. Note also that these numbers refer to PFRs in all known protein isoforms according to ENSEMBL v72. While the results for all these PFR-Drug pairs can be browsed at http://www.cancer3d.org [17], in this manuscript we only discuss results obtained for the largest isoform of each protein (S1 Fig.). A similar protocol to assign protein functional regions was used in our previous publication on identifying domain cancer drivers [13].

### Identification of PFR perturbations correlating with drug activity

As explained above, e-Drug looks for PFRs that, when mutated, correlate with drug activity of the different drugs. We divided the cell lines into those that have a coding missense mutation in the region being studied and those that do not. We then performed a Wilcoxon test comparing the drug activity of each compound in the two groups and kept those with a p-value below 0.01. Finally, for those gene regions associated to a certain drug, the activity of the cell lines mutated in the region of interest was compared to the activity of cell lines mutated in other regions of the gene. By doing this, regions that are significantly different from the rest of the gene were identified. In this case, since the number of cell lines in both groups is lower and fewer tests were performed, a significance threshold of 0.05 instead of 0.01 was established. We considered as true positives those PFR that passed both thresholds and that are not in proteins that show an association (p<0.01) with the same drug at the whole-protein level (S1 Fig.). Note that the analysis is performed independently for each PFR. In the case that a protein contains two overlapping regions, e-Drug will handle each one of them independently and return their corresponding results.

### Statistical significance analysis

One of the problems that arise when analyzing PFRs instead of whole proteins is that the statistical power of the sample decreases significantly, as (I) there are less cell lines with mutations in the individual regions and (II) the number of correlations being tested increases, making multiple-testing corrections more stringent. To overcome these limitations and decrease the number of false positives among our associations we require three different thresholds for an association to be considered positive (see Fig. 1 and S1–S3 Figs.). First, the p value of comparing the activity of the drugs between cell lines with mutations in the PFR against those without them has to be below 0.01. This left us with 350 potential PFR-drug pairs identified in the CCLE data. Then, we repeated the analysis at the protein level and removed all the pairs that were also identified there (p<0.01, n = 102, Fig. 1f). Finally, for the remaining 248 pairs, we compared the drug activity of the cell lines with mutations in the PFR against cell lines with mutations in other regions of the same protein.

### Protein expression data from TCPA

Expression data for 461 different proteins in 93 cancer cell lines was downloaded from the TCPA website on 12/11/2013. Cell line names used in TCPA were manually mapped to CCLE when automated mapping was not possible.

In order to find proteins with altered expression or phosphorylation levels in cell lines with mutations in PFRs of interest cell lines, we grouped them according to the mutation status of such PFRs and compared the expression levels in each group using a Wilcoxon test. To find proteins whose expression correlated with the activity of anticancer drugs we performed a Pearson correlation test using R.

### TCGA survival analysis

We have downloaded both, clinical and mutation data, for the 3,205 tumors described in the pan-cancer analysis of the TCGA. We then filtered out data from patients that had not been treated with any of the drugs included in the CCLE. Since most drugs included in the CCLE are still in under clinical research, we only had enough patients to analyze 2 different drugs: paclitaxel (n = 778) and Irinotecan (n = 58). Each of these subsets of patients have then been classified in 3 groups: those that have a mutation in a PFR that, according to our analysis, increases resistance to the drug used to treat them, those with mutations in other regions of the same genes and those with no mutations in these genes.

We have limited our analysis to gene-regions associated with lower drug activity because there are more such regions as compared to regions associated with increased activity. As a result very few patients in the TCGA dataset carry mutations in the former type of regions and have been treated with the matching drug. The survival analysis has been performed using the “Survival” package for R.

### Protein–drug interaction data

It would be natural to expect that proteins that are associated with drug phenotypes might be enriched in either drug targets or their partners. To test this hypothesis, we downloaded the STITCH database that contains information on protein–chemical interactions. We then retrieved for each drug its known protein interactions and compared the overlap of proteins on this list with the proteins that showed an association with that same drug according to our analysis with the Fisher test. We performed the analysis using three different thresholds for the protein-drug interaction score as reported in STITCH: 700, 800 and 900. We also extended the analysis to (a) proteins interacting with drug targets (according to either HPRD, BioGRID, MINT, or DiP) and to (b) proteins that bind chemicals with a similar structure. We defined these drug-like chemicals as those that have a Tanimoto 2D similarity score with the drug over 0.70. We calculated the Tanimoto scores with the R package RCDK.

## Supporting Information

S1 Fig **Distribution of the p values for all the pairs considered for analysis.** (a) When taking into account all the protein isoforms expressed in each gene there are 739,152 possible PFR-Drug pairs (blue region). In order to limit the number of regions considered for the study we only considered PFRs located in the largest isoform of each gene, leaving us with 202,417 possible pairs (green region). However, only 99,758 had at least 2 mutations in CCLE, which is the minimum number that we considered to start the analysis (red circle). (b) Distribution of p values for all the analyzed pairs. As expected, most pairs have a p value around 1, whereas only 405 are below the 0.01 threshold (vertical red dashed line). (c) The distribution of mutations across the different PFR-Drug pairs follows a power-like distribution, as most pairs have less than 20 mutations, but a few pairs have over 150. (d) Relationship between number of mutations in each pair and the observed p value. As expected, as the number of mutations in each PFR-Drug pair is not correlated with the number of mutations, however, there are no pairs with p values <0.01 (horizontal red dashed line) and less than three mutations.
(TIF)

S2 Fig **Protein functional regions within genes that are also statistically significant are considered false positives.** (a) Cell lines with mutations in the kinase domain of PRKG2 (between red dashed lines) show similar sensitivity towards 17-AAG than cell lines with mutations in the rest of the protein. (b) While there cell lines with mutations in the Kinase domain of PRKG2 show statistically significant lower 17-AAG activity (p<0.004), the signal is also preserved (p<2-e6) at the whole gene level. This suggests that this PFR is associated to this drug because it belongs to PRKG2, not because there is something specific to the PFR.
(TIF)

S3 Fig **Protein regions that show differences when compared to the rest of the protein are considered true positives.** (a) The intrinsically unstructured region (IUR) between positions 334 and 699 (red dashed lines) in AFF4 is associated with increased sensitivity towards the MEK inhibitor PD-0325901. (b) The difference is statistically significant not only when compared to cell lines with no mutations in AFF4 (p<0.003), but also when compared to cell lines with mutations in other regions of the same protein (p<0.002).
(TIF)

S4 Fig **Drug-PFR containing proteins do not usually interact with the drug or the drug's targets.** We checked the overlap between PFR-containing proteins and each drug's targets (top panel) or proteins interacting with them (second panel from the top). Only PFRs associated with AZD6244 were enriched in drug targets (p<0.005, horizontal red dashed line). Extending the search to chemical matter with similar structure to that of each drug (Tanimoto score >70) yielded similar results (two bottom panels).
(TIF)

S1 Table **PFR-Drug associations and links to Cancer3D.**
(XLS)

S1 Supporting Material **Extended analyses and supporting figures.** This file contains extended details about the p-values distribution, the different p-value thresholds used in our analysis, information about the protein-drug experiment as well as S1–S4 Figs.
(DOCX)
