# Supplementary figures and images for: Analysis of Individual Protein Regions Provides Novel Insights on Cancer Pharmacogenomics
Source: PLoS Comput Biol. 2015 Jan 8;11(1):e1004024. doi: 10.1371/journal.pcbi.1004024 (PMC4287345; doi:10.1371/journal.pcbi.1004024)

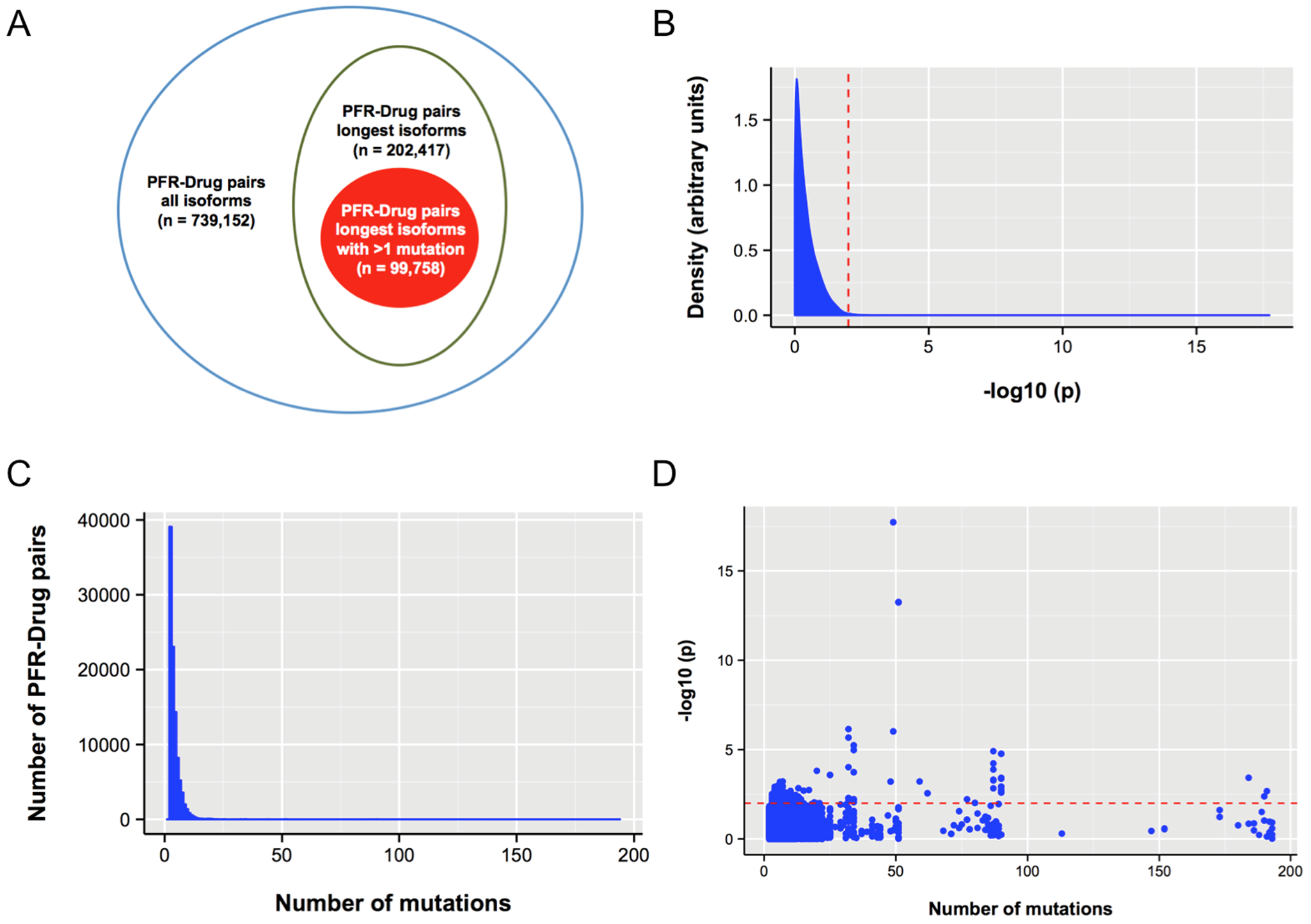

Supplement: S1 Fig — Distribution of the p values for all the pairs considered for analysis. (a) When taking into account all the protein isoforms expressed in each gene there are 739,152 possible PFR-Drug pairs (blue region). In order to limit the number of regions considered for the study we only considered PFRs located in the largest isoform of each gene, leaving us with 202,417 possible pairs (green region). However, only 99,758 had at least 2 mutations in CCLE, which is the minimum number that we considered to start the analysis (red circle). (b) Distribution of p values for all the analyzed pairs. As expected, most pairs have a p value around 1, whereas only 405 are below the 0.01 threshold (vertical red dashed line). (c) The distribution of mutations across the different PFR-Drug pairs follows a power-like distribution, as most pairs have less than 20 mutations, but a few pairs have over 150. (d) Relationship between number of mutations in each pair and the observed p value. As expected, as the number of mutations in each PFR-Drug pair is not correlated with the number of mutations, however, there are no pairs with p values <0.01 (horizontal red dashed line) and less than three mutations. (TIF) [file pcbi.1004024.s001.tif]

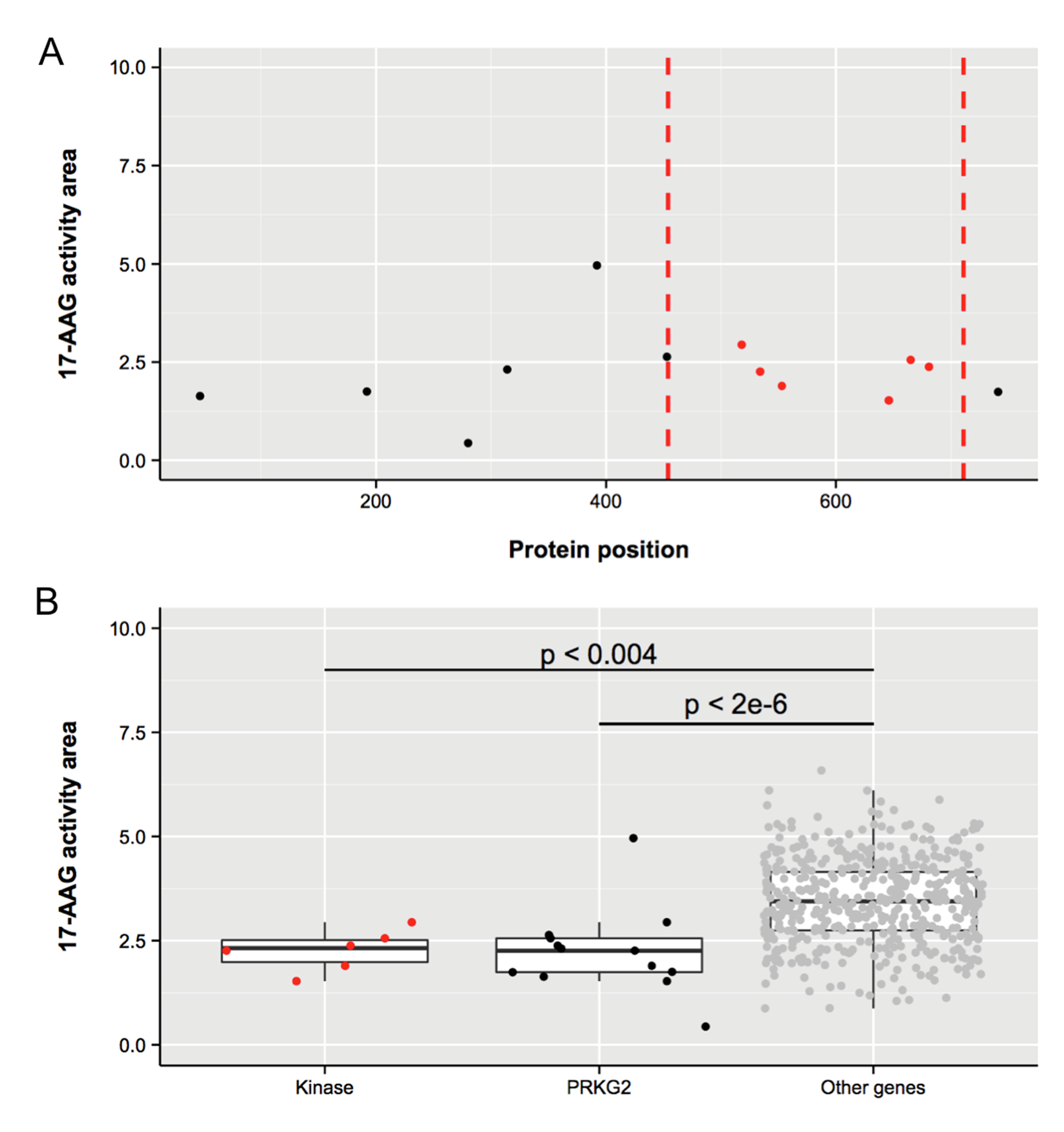

Supplement: S2 Fig — Protein functional regions within genes that are also statistically significant are considered false positives. (a) Cell lines with mutations in the kinase domain of PRKG2 (between red dashed lines) show similar sensitivity towards 17-AAG than cell lines with mutations in the rest of the protein. (b) While there cell lines with mutations in the Kinase domain of PRKG2 show statistically significant lower 17-AAG activity (p<0.004), the signal is also preserved (p<2-e6) at the whole gene level. This suggests that this PFR is associated to this drug because it belongs to PRKG2, not because there is something specific to the PFR. (TIF) [file pcbi.1004024.s002.tif]

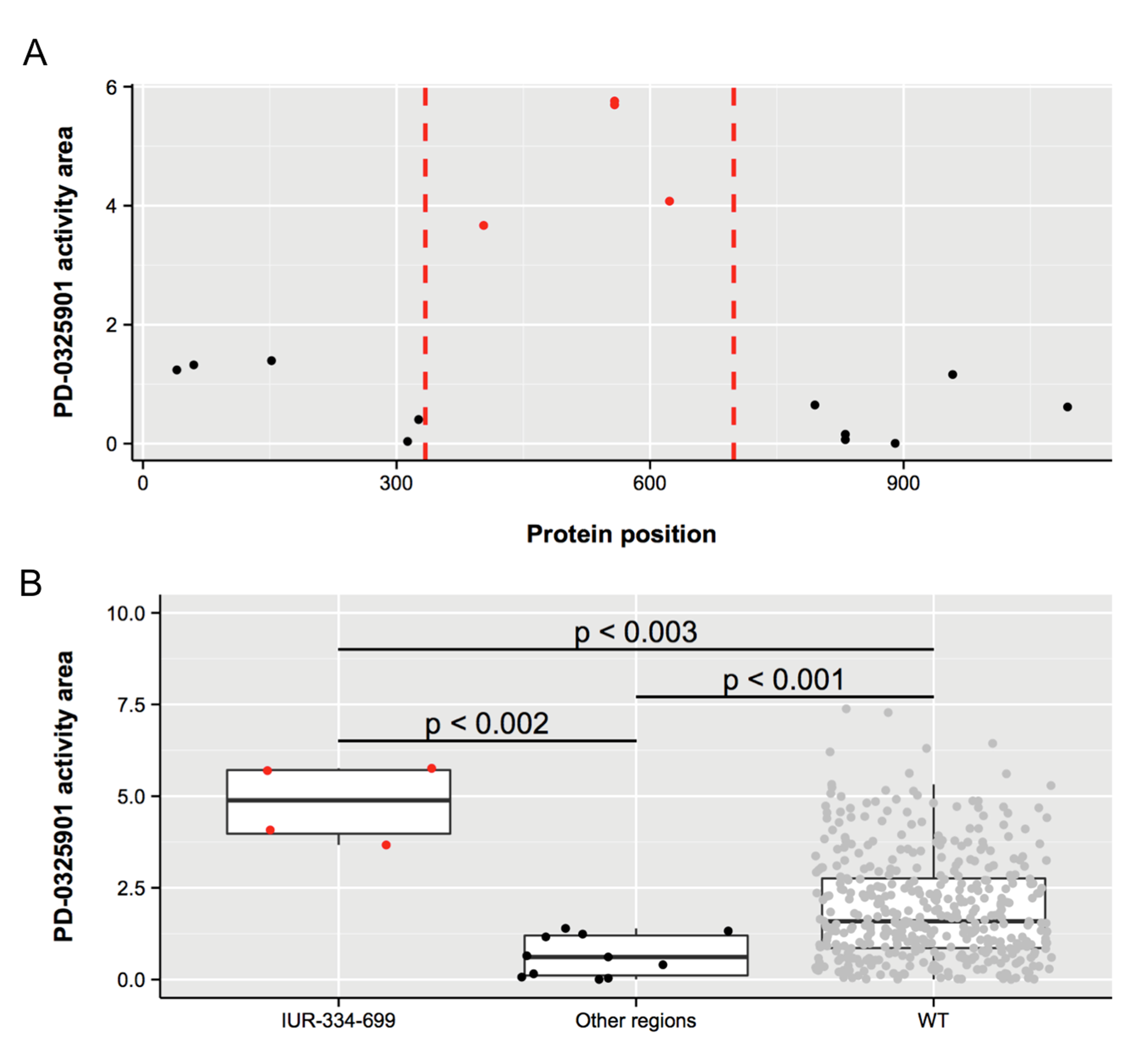

Supplement: S3 Fig — Protein regions that show differences when compared to the rest of the protein are considered true positives. (a) The intrinsically unstructured region (IUR) between positions 334 and 699 (red dashed lines) in AFF4 is associated with increased sensitivity towards the MEK inhibitor PD-0325901. (b) The difference is statistically significant not only when compared to cell lines with no mutations in AFF4 (p<0.003), but also when compared to cell lines with mutations in other regions of the same protein (p<0.002). (TIF) [file pcbi.1004024.s003.tif]

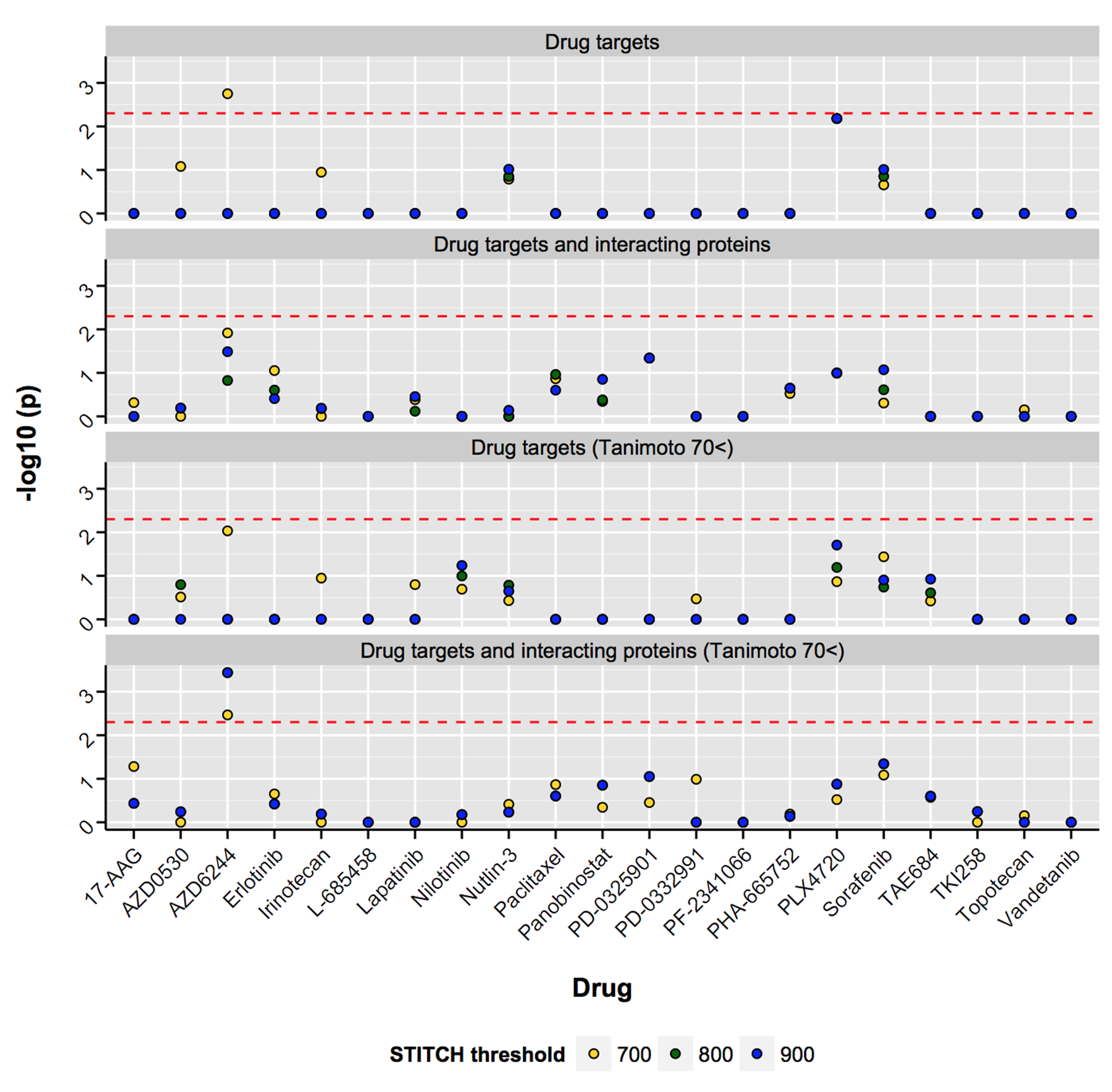

Supplement: S4 Fig — Drug-PFR containing proteins do not usually interact with the drug or the drug's targets. We checked the overlap between PFR-containing proteins and each drug's targets (top panel) or proteins interacting with them (second panel from the top). Only PFRs associated with AZD6244 were enriched in drug targets (p<0.005, horizontal red dashed line). Extending the search to chemical matter with similar structure to that of each drug (Tanimoto score >70) yielded similar results (two bottom panels). (TIF) [file pcbi.1004024.s004.tif]
