# Supplementary material for: Analysis of Individual Protein Regions Provides Novel Insights on Cancer Pharmacogenomics
Source: PLoS Comput Biol. 2015 Jan 8;11(1):e1004024. doi: 10.1371/journal.pcbi.1004024 (PMC4287345; doi:10.1371/journal.pcbi.1004024)
Supplement: S1 Supporting Material — Extended analyses and supporting figures. This file contains extended details about the p-values distribution, the different p-value thresholds used in our analysis, information about the protein-drug experiment as well as S1–S4 Figs. (DOCX) [file pcbi.1004024.s006.docx]

**Supporting Material for the *PLOS Computational Biology* submission:**

**“Exploring protein regions to find novel insights on cancer pharmacogenomics”**

Eduard Porta-Pardo^1^ and Adam Godzik^1*^

^1^Bioinformatics and Systems Biology Program, Sanford-Burnham Medical Research Institute, 10901 North Torrey Pines Road, La Jolla, CA 92037, USA

^*^To whom correspondence should be addressed (adam@godziklab.org)

**Introduction**

In this supporting material we provide more details about the e-Drug results when analyzing data from the Cancer Cell Line Encyclopedia [1], particularly about the distribution of p values (Supporting Figures 1-3), and an extension of the analysis regarding the protein-drug interactions using data from STITCH [24] (Supporting Figure 4).

**Global view of the results obtained using e-Drug**

One of the problems facing most “-omics” technologies and analyses is how to deal with the multiple protein isoforms expressed by the same gene. A commonly used approach consists in analyzing only the longest isoform of each gene [13]. In our analysis, while we have analyzed the different protein functional regions of all the annotated isoforms in ENSEMBL v72, and one can consult these results at http://www.cancer3d.org [17], in the manuscript we only discuss results obtained for the longest isoform of each gene. This reduces the number of possible PFR-Drug pairs from 739,152 to 202,417. Furthermore, only 99,758 of such pairs had at least 2 mutations (Supporting Figure 1a), which is the minimum number that we established to perform the analysis. Given that we also established a p value threshold at 0.01 to consider a PFR-Drug pair a possible positive, one would expect to observe, solely by chance, ~1,000 pairs below this threshold, yet we only found 405 of such PFR-Drug pairs. The reason is that only PFR-Drug pairs with at least 3 mutations can achieve a p value below the 0.01 threshold in the CCLE dataset reducing the number of possible pairs to only 60,680.

We then faced the problem of distinguishing the true positives from the true negatives among these 405 pairs with p < 0.01. In this scenario it is recommendable and standard practice to use multiple testing correction tools, however since the number of cell lines with mutations in each PFR is usually low (Supporting Figure 1c), most p values after applying such tools are very high.

In this scenario, we suspected that by applying traditional multiple testing algorithms we would have a high number of false negatives. To circumvent this problem, instead of any standard multiple testing approach, we used 2 additional thresholds based on biological insights that allowed us to discard most of the likely false positives in the list of 405 candidate pairs.

The first threshold consists in removing from the list all the PFR-Drug pairs in proteins that have a signal also at the whole-protein level. This removes all the domains that belong to proteins where, regardless of the region, mutations cause a change in the sensitivity towards a drug (Supporting Figure 2). These are likely PFRs that are associated to a drug because they are located in a protein that is important for the response towards that drug, not because the PFR itself is relevant. This first threshold removed 157 PFR-Drug pairs of the original 405.

We then reanalyzed the data for the remaining 248 as we still faced another problem. There are some cases where most mutations occur in a specific domain. In that scenario, we do not know if the positive signal comes from the PFR or the gene levels. To further curate the list of candidate PFR-Drug pairs, we compared the drug activity in cell lines with mutations in the candidate regions with that of cell lines with mutations in other regions of the same protein (Supporting Figure 3). If we observed a difference statistically significant also in this comparison (p < 0.05) we declared the pair a true positive, as we believe we strongly ruled out the possibility that the association comes from the gene level or anywhere else than the PFR being studied. This yielded the final list of 171 PFR-Drug pairs that were further analyzed.

**Physical interactions between drugs and PFRs**

We wondered whether proteins containing PFRs associated to changes with drug activity would also be targets of the drugs themselves. If that were the case it would be indicative as to why we observe that association. To explore this possibility we used drug-protein interaction data from STITCH [24] and compared, for each drug, the number of proteins that contain drug-altering PFRs and at the same time physically interact with the drug, with the number that would be expected by chance given the number of drug targets and the number of PFR-containing proteins (Supporting Figure 4). Surprisingly, only PFRs for one of the 19 drugs explored, AZD6244, were enriched in drug targets (p < 0.005). To further explore this idea we tried varying the interaction score from STITCH between 700 and 900, but this did not alter the results.

We then expanded the analysis to proteins interacting with the drug targets, as this is another obvious possibility that could explain why a PFR is associated to a specific drug. Nevertheless, none of the drugs showed a statistically significant enrichment in this experiment.

Finally, we tested the possibility that PFR-containing proteins might be interacting with other molecules with similar structures as the drugs. This would suggest that the protein might actually interact with the drug, even if the interaction is not included in STITCH. In order to do that we repeated the two experiments extending the search to any protein interacting with molecules that had a Tanimoto score above 70 to the specific drug, but, again, only AZD6244 showed statistically significant associations.
